# Advanced Molecular Solutions for Cancer Therapy—The Good, the Bad, and the Ugly of the Biomarker Paradigm

**DOI:** 10.3390/cimb46030109

**Published:** 2024-02-22

**Authors:** Dumitru Andrei Iacobas

**Affiliations:** Laboratory of Personalized Genomics, Undergraduate Medical Academy, Prairie View A&M University, Prairie View, TX 77446, USA; daiacobas@pvamu.edu; Tel.: +1-936-261-3086

Identifying the most effective actionable molecules whose “smart” manipulation might selectively kill/slow down/stop the proliferation of cancer cells, with few side effects on the normal cells of the tissue, was for decades the single major objective of countless investigators. This Special Issue (SI), a continuation of the *previous Current Issues in Molecular Biology* SI “Molecules at Play in Cancer” [1], aimed to present the latest developments in the molecular solutions for cancer therapy, and the ways of personalizing the treatment to the individual characteristics of the patient. The authors have contributed their best efforts, either by performing accurate experiments, reanalyzing publicly accessible genomic datasets (including The Cancer Genome Atlas (TCGA) [2]), or writing comprehensive reviews of the literature. Among the interesting proposed solutions, of note are: the targeted delivery of chimeric antigen receptor into T cells via CRISPR [3], the metabolic silencing via methionine-based amino acid restriction [4], the inhibition of ERK5 [5], the activation of *ADRA2A* [6], and the aspirin treatment of breast cancer [7].

Most articles dealing with molecular mechanisms that should be activated or inhibited to destroy the cancer cells either directly, (e.g., [5]), or by increasing the efficacy of immuno- [3,8], chemo- [6], and radiotherapy were aligned with the main stream “biomarker paradigm” (BMP). For a long time, the vast majority of investigators and clinical oncologists have believed that the mutation and/or altered expression of certain genes, called “biomarkers”, are responsible for triggering cancerization. Moreover, it was hoped that restoring the normal status of such gene biomarkers would provide the natural anti-cancer therapy. The efficacy of various biomarker-oriented gene therapies was tested on both standard human cancer cell cultures and animal models. 

However, let us have a candid discussion about how cancer biomarkers have been discovered, what their real values are for diagnostic and therapy, and how reliable their testing is on animal models and human cell cultures. Decades of work at almost all stages of both experimental and theoretical genomic research using numerous types of platforms entitle and oblige me to take the risky enterprise of discussing “the good, the bad and the ugly” of this paradigm. Studies of my lab-profiled cell cultures and tissues from surgically removed human tumors, as well as a wide variety of cells and tissues from animal (mouse, rat, rabbit, dog, chicken) models of human diseases. In addition to optimizing the wet protocols, we have introduced the Genomic Fabric Paradigm and developed advanced mathematical algorithms and computer software to analyze the genomic data. 

The GOOD (BMP promises). Cancer is a multi-factorial disease regulated by an enormous number of widely diverse favoring conditioners, understanding of which is far from being complete. Therefore, relying on a few gene biomarkers (even with each of them presenting several variants [9]) is a considerable simplification of the diagnostic process. One of the greatest advantages of BMP is that (in theory) it provides molecular explanations of the origin of various cancer forms. 

Several test kits (e.g., [10,11,12]) have been developed and are currently in use for the genomic detection of an existing, particular cancer form or the perspective of a tissue that will undergo sooner or later a malignant transformation. For instance, the “Invitae Multi-Cancer Panel” [10] of 70 genes specifically designed for heritable germline mutations in blood, saliva, or buccal swab specimens sequences *BRCA1* and *BRCA2* to detect hereditary breast and ovarian cancer syndrome. The “nCounter PanCancer IO 360™ Panel” [11], a unique 770 gene expression assay looking for the transcriptomic signatures of various cancer forms, which provides a number of cancer risk scores. “TissueScan™ Cancer and Normal Tissue cDNA Arrays” [12] were developed for differential gene expression analysis by comparing patient samples with pathologist-verified cancer and normal tissues.

The biomarkers are also considered legitimate targets for cancer gene therapy, offering significant economic advantages in the large-scale production of biomarker-manipulating medicines that should be prescribed to all persons affected by the same cancer form. In contrast, the economic incentives of the precision medicine that tries to tailor the treatment to the patient’s characteristics are still limited, especially for low-income countries [13,14]. Owing to these (mostly desired rather than real) benefits, BMP serves to standardize the oncological recommendations and procedures (e.g., [15,16]). 

To resume, the very important BMP (believed) GOODs are that:

(1) it provides a simple molecular mechanistic explanation of cancerization in any human and animal regardless of race/strain, sex, age, or any other personal and environmental characteristic;
(2) it is the theory behind organizing the genes in functional pathways by the very popular software: Ingenuity Pathway Analysis [17], DAVID [18], and KEGG [19];
(3) it provides the reason for developing universal assays to detect the existing cancer of a particular form and/or estimating the chances of future cancerization for any person;
(4) it stimulated the development of animal models and engineered cell cultures to mimic various forms of human cancer, validate their genetic etiology, and test gene therapy;
(5) it is the basis of designing therapeutic solutions that target the molecular mechanisms of cancerization;
(6) it supports the standardization of the oncological procedures by the National Comprehensive Cancer Network (e.g., [20]);
(7) it has been adopted by the vast majority of genomic researchers (as of 6 January 2024, PubMed [21] listed 421,759 “cancer biomarker” and 957,483 “cancer genetic etiology” publications);
(8) it benefits from the most generous research funding by public and private agencies and is of major interest for the pharma industry.

The BAD (BMP reality). According to the 39.0 release (12 April 2023) of the NIH-NCI Harmonized Cancer Datasets [22], almost every single gene was found as mutated in at least one case of almost every form of cancer and every form of cancer exhibited mutations in almost all genes. So, what about the specificity of the biomarkers? 

Nonetheless, together with the blamed biomarker(s), hundreds of other genes appear as mutated and/or regulated when comparing tissues from cancer stricken and healthy persons; however, their potential contributions to the cancer phenotype are mostly ignored by the BMP users. One reason for disregarding the other altered genes might be that their combination is practically never exactly repeated among patients and changes (slowly but steadily) in time for the same person. 

It is legitimate to ask how the developers of the cancer test kits determined and validated the predictive values of the transcriptomic signatures since not 770 but only 30 genes that can be up-/down-/not regulated form over 2 × 10^14^ distinct combinations (many more than living humans). 

Most biomarkers were identified through meta-analyses that compared DNA sequences and/or RNA expression profiles from tissues of cancer patients and healthy counterparts. In order to increase the statistical significance of the biomarkers, numerous analyses covered data collected by several laboratories (using sometimes different wet protocols and/or equipment) from large and in many cases heterogeneous (as in race, sex, age) populations. Thus, beyond possible human errors, protocol differences, and the normal technical noise of the platform, the data were biased by the distinct cancer prevalence among races, sexes, and age groups (to name just a few favoring factors) [23]. Owing to the dispersions of the gene expression levels among cancer-stricken and healthy persons, the difference between the mean values of the two distributions is most likely smaller than between the extreme values within each distribution. Thus, two healthy persons may have larger transcriptomic differences than a healthy and a cancer-stricken person (Figure 1).

Nonetheless, the tumors are heterogeneous, harboring cell subpopulations with distinct genotypes and phenotypes, and it is a very slim probability that in the large repository, the profiled samples have been collected from exactly the same type of clones and/or region of the tissue (affected by the microenvironment) from different individuals. Intra-tumor genetic and transcriptomic heterogeneity was reported by many groups (e.g., [24,25,26,27,28]). In our gene expression studies (e.g., [29,30]), we found large differences even between equally graded cancer nodules from the same tumor. So, what do “genetic etiology” and “transcriptomic signature” (both deduced by comparing the average cancer patient with the average healthy counterpart) stand for when genes from different locations within the same tumor exhibit different mutations and expression regulations? Therefore, the best comparison is not between the tissues of the average cancer-stricken person and average healthy counterpart, but between the cancer nodule(s) and surrounding cancer-free tissue from the same tumor. This strategy has already been adopted by several laboratories (e.g., [29,30,31,32,33,34]).

About the BMP-based therapy: as selected from the most frequently altered genes in large populations of people harboring similar cancer forms, biomarkers appear as the least protected by the homeostatic mechanisms, an indication of their low importance. Therefore, restoring their normal status might be of little consequence.

In summary, the BMP BADs are:

(1) low diagnostic specificity owing to the large number of cancer forms harboring the same biomarker;
(2) insufficient diagnostic sensitivity, numerous cases missing the alleged biomarker (e.g., [35]);
(3) disconsiders major personal favoring factors of the patient, including race, sex, age, diet, and environmental factors, such as exposure to radiation, toxins and stress;
(4) differences between healthy persons might be larger than between the average healthy and the average cancer-stricken persons;
(5) disregard of the contributions of the many other genes whose sequences and/or expression levels are altered (even in not repeatable combination) in the cancer of each individual;
(6) BMP-based functional pathways do not discriminate with regard to race, sex, and age, do not change with the cancer progression and/or in response to external stimuli and treatments, and are reduced to unique gene networking;
(7) it selects low cell players for gene therapy.

The UGLY (BMP justification). Whilst even a germline mutation is supposed to be present in all cells, only a part of them evolves into a cancer phenotype, indicating the importance of the local environment. Moreover, most cancers were not inherited but occur and disappear spontaneously in some cells of otherwise homo-cellular tissues. 

A very important issue is related to the experimental resolutions of both sequencing and expression studies. Although the single-cell sequencing technology discriminates the genomic characteristics of cell subpopulations with distinct phenotypes, it is still unable to quantify gene alterations within a single cell. Not only does it take a critical number of neighboring cells harboring synchronously the same mutation/expression regulation to start developing a tumor but this is also necessary to be detected by the experimental platform. 

Despite the strict control exerted by the cellular homeostatic mechanisms, both DNA replication and transcription are affected by errors caused by the stochastic nature of the involved chemical reactions. The average one of each of a 1000 nucleotides being mutated at any time (i.e., over 3 mil spontaneous mutations in every cell genome) makes it practically impossible to find an unmutated gene in any cell of the human body. The expected number of mutations in a gene equals the number of its composing kilo bases but there is a negligible overlapping of the mutations among the cells of the tissue, making most of them impossible to detect. 

Moreover, the expression level of each gene fluctuates around the cell-cycle dependent value to provide the needed rate of protein synthesis and, although correlated, fluctuations are not synchronized among all cells of the tissue. Therefore, the transcriptome is not homogeneous across the tissue and the detection of significantly regulated genes (and implicitly the transcriptomic signature) depends on the selection of the profiled regions in the compared tissues. Not to mention that the arbitrarily introduced absolute fold-change cut-off (e.g., 1.5×) for a gene to be considered as significantly up-/down-regulated is too stringent for stably expressed genes across biological replicas and low-noise platforms and too lax for variably expressed genes and noisier platforms.

The experimental validation of both BMP-based diagnostic and targeted therapy is disputable owing that manipulating the sequence and/or the expression level of one gene has ripple effects on hundreds of other genes. There is no way of dissecting the contribution of the biomarker from those of the other genes in any genetically engineered animal model or cell line.

Therefore, the BMP UGLY aspects are related to the limited resolution and high technical noise of the actual genomic platforms, together with the impossibility of experimentally validating the functional roles of the biomarkers and the benefits of the biomarker-targeting therapy.

The continuing development of the next generation sequencing and the unlimited power of AI algorithms will soon decrease the need of the oversimplified BMP looking for genes universally responsible for a particular cancer form in any human. The clinical oncologists are already moving from the “fit-for-all” model to personalized gene therapies. The spectacular advancements in gene-editing technologies and the economic incentives are expected to convince the pharma industry to start producing shelf-ready constructs to manipulate the master regulators identified for each cancer patient (e.g., [29,30]). 

## Figures and Tables

**Figure 1:**
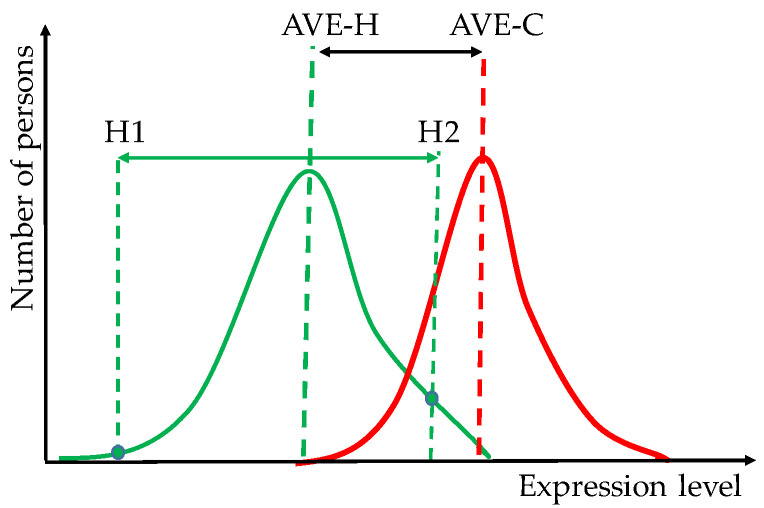
Potential distributions of the expression levels of a hypothetical gene biomarker within healthy (H) and cancer-stricken (C) persons. Note that the difference between the expression levels in two healthy individuals (H1 and H2) may be larger than that between the average healthy (AVE-H) person and the average cancer-stricken (C) person.
